# Cardiac and Metabolic Impact of Functional Foods with Antioxidant Properties Based on Whey Derived Proteins Enriched with Hemp Seed Oil

**DOI:** 10.3390/antiox9111066

**Published:** 2020-10-30

**Authors:** Teresa Pasqua, Carmine Rocca, Francesca Romana Lupi, Noemi Baldino, Daniela Amelio, Ortensia Ilaria Parisi, Maria Concetta Granieri, Anna De Bartolo, Arturo Lauria, Marco Dattilo, Ida Daniela Perrotta, Francesco Puoci, Maria Carmela Cerra, Domenico Gabriele, Tommaso Angelone

**Affiliations:** 1 Laboratory of Cellular and Molecular Cardiovascular Pathophysiology, Department of Biology, E. and E.S. (Di.B.E.S.T.), University of Calabria, 87036 Rende (CS), Italy; teresa.pasqua@unical.it (T.P.); carmine.rocca@unical.it (C.R.); mariaconcetta.granieri@unical.it (M.C.G.); anna.de_bartolo@unical.it (A.D.B.); d.marco1091@hotmail.it (M.D.); 2 Laboratory of Rheology and Food Engineering, Department of Information, Modeling, Electronics and System Engineering (D.I.M.E.S.), University of Calabria, 87036 Rende (CS), Italy; nbaldino@dimes.unical.it (N.B.); d.gabriele@unical.it (D.G.); 3 Laboratory of Organ and System Physiology, Department of Biology, E. and E.S (Di.B.E.S.T.), University of Calabria, 87036 Rende (CS), Italy; daniela.amelio@unical.it (D.A.); maria_carmela.cerra@unical.it (M.C.C.); 4 Department of Pharmacy, Health and Nutritional Sciences, University of Calabria, 87036 Rende (CS), Italy; ortensiailaria.parisi@unical.it (O.I.P.); francesco.puoci@unical.it (F.P.); 5 Macrofarm s.r.l., c/o Department of Pharmacy, Health and Nutritional Sciences, University of Calabria, 87036 Rende (CS), Italy; 6 ASL San Marco Argentano (CS), Veterinary Medicine Section, 87018 San Marco Argentano (CS), Italy; arturo.lauria@tiscali.it; 7 Centre for Microscopy and Microanalysis, Transmission Electron Microscopy Laboratory, Department of Biology, E. and E.S. (Di.B.E.S.T.), University of Calabria, 87036 Rende (CS), Italy; ida.perrotta@unical.it; 8 National Institute of Cardiovascular Research (I.N.R.C.), 40126 Bologna (ER), Italy

**Keywords:** cardioprotection, whey-derived proteins, hemp seed oil, functional foods, nutrition, rheological approach

## Abstract

The impaired ability to feed properly, evident in oncologic, elderly, and dysphagic patients, may result in malnutrition and sarcopenia. Increasing the consumption of dietary proteins by functional foods and enriching their composition by adding beneficial nutrients may represent an adjuvant therapy. We aimed to evaluate the safety and the positive effects of a standard diet (SD) supplemented with whey-derived protein puddings (WDPP), with appropriate rheological properties, and hemp seed oil (HSO), rich in polyphenols. Rats were assigned to SD, WDPP, WDPP plus hemp seed oil (HSOP), and HSO supplemented diets for eight weeks. “Anthropometric”, metabolic, and biochemical variables, oxidative stress, tissue injury, liver histology, and cardiac susceptibility to ischemia/reperfusion were analyzed. All the supplementations did not induce significant changes in biochemical and metabolic variables, also in relation to glucose tolerance, and livers did not undergo morphological alteration and injury. An improvement of cardiac post-ischemic function in the Langendorff perfused heart model and a reduction of infarct size were observed in WDPP and HSOP groups, thanks to their antioxidant effects and the activation of Akt- and AMPK-dependent protective pathways. Data suggest that (i) functional foods enriched with WDPP and HSOP may be used to approach malnutrition and sarcopenia successfully under disabling conditions, also conferring cardioprotection, and that (ii) adequate rheological properties could positively impact dysphagia-related problems.

## 1. Introduction

Chronic diseases and severe illness are commonly associated with malnutrition and sarcopenia [1]. Malnutrition is a pathological condition caused by an energy deficiency, and/or by a relative absence of protein and other nutrients, resulting in negative clinical outcomes because of the altered physiological composition and function of tissues and organs [2]. As a major negative effect, malnutrition often leads to sarcopenia, generally considered to be a geriatric syndrome but recently identified as an independent pathological condition also detectable in young people [3]. Sarcopenia is defined as a state in which, in a human being of a specific age, gender and race, a less-than-expected muscle mass can be detected [4]. It is a clinical syndrome characterized by a decline in physical function, since the decreased skeletal muscle mass produces an impaired muscle strength [5]. If untreated, sarcopenia may deeply impact quality of life and develop into a serious burden from personal, social, and economic points of view [6]. Many times, malnutrition and sarcopenia are present together. Indeed, a sign of malnutrition is represented by the unintentional loss of weight that, in turn, causes a loss of muscle mass resulting in an increased risk of functional impairment [1]. Both negatively affect the outcome in clinical healthcare and are strongly related to an increased risk for cardiometabolic diseases [7].

A higher incidence of malnutrition is visible in patients affected by dysphagia [8,9,10,11], that may result from oldness, and/or neurological, neurodegenerative, oncologic, as well as head and neck health problems [12]. In this case, patients show a reduced consumption of fluids and foods, especially according to their consistency and texture, which increases the risk of unbalanced feeding [11,13]. Safe swallowing is critically determined by the oropharyngeal phase, when the bolus is propelled by the tongue from the oral cavity to the oropharynx [12]. Under physiological conditions, a fine coordination among muscles and nerves generates a dynamic separation between the alimentary and the respiratory streams. This regulatory mechanism is weakened in people suffering from dysphagia [14]. It is known that swallow-respiratory temporal coordination is also determined by the food bolus consistency [12] and that swallowing processes can be analyzed from a fluid kinematics/dynamics point of view [15]. Therefore, food bolus rheology (i.e., food bolus flow properties) is fundamental in relation to dysphagia and to the possibility of supporting the development of useful nutritional tools able to counteract the consequences of the pathology. Indeed, starting from the assumption that rheology is interested in the study of matter deformation and flow, it is easy to find a deep connection with swallowing diseases [12]. Of note, recent findings point to food rheology and food texture modification as promising therapeutic strategies for dysphagic patients ([16] and references therein, [17] and references therein).

A proper nutrition, particularly in relation to protein intake, plays an important role in treating the loss of muscle mass [18,19] and references therein. Thus, it is possible that functional foods enriched with proteins, and with appropriate rheological properties, may successfully be used to approach disease-induced malnutrition and sarcopenia. In fact, it is known that functional foods contain compounds with beneficial preventive actions and appropriate nutritional effects, also able to mitigate some pathological conditions [20].

Starting from this information, by using the rat as an experimental model, we aimed to collect preliminary data about the safety and the possible beneficial effects of a standard diet supplemented with whey-derived proteins puddings (WDPP), or with WDPP plus hemp seed oil (HSOP), or with hemp seed oil alone (HSO). The supplementation with WDP can contribute to maintain skeletal muscle mass [21,22] while the HSO supplementation can mitigate the cardiovascular risk related to diseased states [23]. Nutritional supplements were designed according to the rheological features that result adequate for dysphagia. In particular, we evaluated the impact of the different diets on (1) “anthropometric” parameters; (2) plasma biochemical profile; (3) liver histology and injury; (4) cardiac susceptibility to ischemia/reperfusion injury.

## 2. Materials and Methods

### 2.1. Animals

The study enrolled male Wistar rats weighting ~250 g (Envigo, Udine—Italy), housed one per cage under controlled conditions of light and temperature. Animals were fed ad libitum and had free access to water. All the protocols were conducted in accordance with the Declaration of Helsinki, the Italian law (D.L. 26/2014), the Guide for the Care and Use of Laboratory Animals published by the US National Institutes of Health (2011) and the Directive 2010/63/EU of the European Parliament on the protection of animals used for science. The project was approved by the Italian Ministry of Health, Rome, and by the ethics review board.

### 2.2. Drugs and Chemicals

Folin-Ciocalteu reagent, sodium carbonate, catechin (CA), 2,2′-diphenyl-1-picrylhydrazyl radical (DPPH), 2,2’-azinobis-(3-ethylbenzothiazoline-6-sulfonic acid) (ABTS), and potassium persulfate were purchased from Sigma-Aldrich (St. Louis, MO, USA). Further, 2,4 dinitrophenylhydrazine (DNPH), 2-thiobarbituric acid (TBA), bovine serum albumin (BSA), butanol-1, butylated hydroxyanisole, diethyl ether, ethylenediaminetetraacetic acid (disodium salt), hexane, streptomycin sulfate, tween-20, and urea were purchased from Sigma Aldrich. Absolute ethanol, ethyl acetate, hydrochloric acid, methanol, and trichloroacetic acid were purchased from Carlo Erba Reagents (Cornaredo, MI, Italy).

### 2.3. Preparation of Diet Supplementation

Puddings were produced following the typical process steps adopted for preparing emulgels [24]. In fact, each phase composing the system was prepared separately and mixed in the final step of the process. The oil phase was based on hemp seeds oil, HSO (Soc. Agr. Antichi Grani, Italy) and, in the case of sample HSOP, soy lecithin (Somercom, Italy). Table 1 shows the composition and the nutritional values of the aqueous phase without the further addition of oil (WDPP), after the addition of oil (HSOP), and of oil alone (HSO).

### 2.4. Aqueous Phase Preparation

The aqueous phase (WDPP) was prepared by suspending Undenatured Whey Powder Isolate (WPI, purity 90% *w/w* with the addition of Soy Lecithin) supplied by Bulk Powders (USA) to distilled water; after a stirring time of 30 min at room temperature, the mixture was then warmed up to 80 °C, using a thermomagnetic stirrer (thermostat Arex, Velp Scientific, Italy). The temperature was held at 80 °C for 1 h to assure the proteins denaturation, and then slowly cooled down to room temperature. The preparation process was the same already described by Lupi et al. [25] for similar systems.

### 2.5. Oil Phase Preparation

The oil phase was prepared by warming the HSO up to 40 °C upon stirring (thermostat Arex, Velp Scientific, Italy), then, after the soy lecithin addition, the system was stirred for further 5 min.

### 2.6. Emulgel Production

The final pudding was then prepared by gently adding the oil phase (HSO) to the aqueous phase (WDPP) at room temperature obtaining samples HSOP (Hemp Seeds Oil Puddings). Homogenization was done thanks to an Ultra Turrax dispersing instrument (UT T50, tool S 50 N-G 45 F, IKA-Werke, Staufen, Germany) gradually increasing the speed of mixing from 1000 rpm up to a maximum of 3500 rpm, until a homogeneous system was obtained.

### 2.7. Rheological Characterization

For the investigated samples Small Amplitude Oscillation Tests were performed at 20 °C: frequency sweep tests were carried out in the Linear Viscoelastic Region (LVR) previously investigated with stress sweep tests (at a constant frequency of 1 Hz). Frequency sweep tests were done at frequencies ranging between 0.1 and 10 Hz, with a stress-controlled rheometer (DSR 500, Rheometric, Piscataway, NJ, USA) adopting a parallel plate geometry (diameter 40 mm, gap 2.0 mm ± 0.1 mm). The rheometer is equipped with a Peltier system for the temperature control (±0.1 °C) of the sample acting under the lower plate. Frequency sweep tests were repeated three times and the results are shown in terms of average values. Error bars are also reported to indicate the standard deviation on each value.

### 2.8. Evaluation of Total Polyphenols Content and Antioxidant Properties

Experimental protocols were performed by recording absorption spectra with a Jasco V-530 UV/Vis spectrometer.

### 2.9. Folin-Ciocalteu Assay

The total polyphenols content of WDPP, HSOP, and HSO was investigated by performing the Folin-Ciocalteu assay according to the literature with slight modifications [26]. For this purpose, 10 mg of each tested item were mixed with 1 mL of distilled water, 1 mL of the Folin-Ciocalteu reagent (0.2 N), and 1 mL of a sodium carbonate solution (7.5% *w/v*). The samples obtained were incubated for 2 h at room temperature and in dark conditions. After filtration, the absorbance was recorded at 760 nm. The amount of total phenolic groups was expressed as mg equivalent of catechin per gram of sample (mg eq CA/g) by using the equation obtained from the calibration curve of the reference compound. The experiments were carried out in triplicate.

### 2.10. DPPH Assay

The scavenging properties of WDPP, HSOP, and HSO toward DPPH radical were evaluated according to the literature with minor modification [26]. For this purpose, different amount of each item (100, 150, 200 and 250 mg) were mixed with 4 mL of an ethanol DPPH solution (188 μM) and 6 mL of ethanol. The samples obtained were incubated in dark conditions for 15 min and, after filtration, the absorbance was measured at 517 nm. The scavenging effect was expressed as inhibition percentage according to Equation (1):

(1) $$inhibition\%=\frac{A_{0}-A_{1}}{A_{0}}\times 100$$

where *A*_0_ is the absorbance of a control prepared in the same experimental conditions, but without any sample, and *A*_1_ represents the absorbance of the tested item. Each experiment was performed in triplicate.

### 2.11. 2,2′-azinobis-(3-ethylbenzthiazoline-6-sulphonic Acid) (ABTS) Assay

With the aim to evaluate the scavenging activity of WDPP, HSOP, and HSO towards ABTS, the radical solution was prepared as previously reported [27]. Different amount of each tested item (100, 150, 200 and 250 mg) were mixed with 1 mL of distilled water and 4 mL of the ABTS radical solution. Then, the obtained samples were incubated in a water bath at 37 °C and in dark conditions for 5 min. After filtration, the absorbance was measured at 734 nm and the results were expressed as inhibition percentage according to Equation (1). The ABTS assay was carried out in triplicate.

## 3. Experimental Groups

The animals were divided into 4 experimental groups (*n* = 6 for each group) feed for 8 weeks as follows:

1. Control group fed with a standard diet (SD, diet 2018: 6.2% kcal fat, 18.6% kcal protein, and 44.2% kcal carbohydrate; Envigo, Udine, Italy)
2. Group WDPP fed with SD supplemented with WDPP (13.33% WPI *w/w*%)
3. Group HSOP fed with SD supplemented with WDPP (13.33% WPI *w/w*%) + HSO (9.5% *w/w*%)
4. Group HSO fed with SD supplemented with 9.5% HSO.

After eight weeks, “anthropometric” parameters were measured, and plasma analysis, morphological analysis, biochemical analysis, and Western blot were performed.

## 4. “Anthropometric” Variables

The measurement of body weight, length and abdominal circumference for each animal was made weekly. Rat length and body mass index (BMI) were calculated as previously reported [28]. After the sacrifice, abdominal and epididymal fat tissue were removed and separately weighed. Liver weight, liver to body weight, and heart weight were measured. Cardiac somatic index (CSI), i.e., the ratio between the heart weight and the animal weight, multiplied by 100, was calculated. The total amount of food and water consumption was recorded daily.

## 5. Biochemical Analysis

### 5.1. Plasma Metabolic Profile

Blood was collected and centrifuged at 4 °C for 15 min at 4000× *g*. Plasma aliquot was frozen (−80 °C) for the subsequent biochemical analyses. Glycemia was determined by using a glucometer (ACCUCHEK, Roche Diagnostics, Germany). Total cholesterol, HDL cholesterol, LDL cholesterol, and triglycerides were determined using a kit from PKL^®^ POKLER ITALY.

### 5.2. Lactate Dehydrogenase (LDH) Activity

The activity of LDH in plasma and cell free extract of liver samples derived from the experimental groups described above was evaluated spectrophotometrically (GENESYS 20 spectrophotometer, Thermo Fisher Scientific), following the method of McQueen [29]. Liver tissues were homogenized in ice cold sucrose (0.25 mol/L) using a homogenizer (T10 basic IKA Ultra-Turrax^®^) to make a 10% homogenate (*w/v*). The homogenate was centrifuged at 10,000× *g* for 15 min at 4 °C and the supernatant was used for LDH activity determination. The protein concentration was determined following the Bradford method using bovine serum albumin (BSA) as a standard. After the incubation of reagents, absorbance at 340 nm was recorded every min for 3 min. The reaction velocity was determined by a decrease in absorbance at 340 nm, resulting from the oxidation of NADH indicative of LDH activity, which was expressed as IU/L and µmoles of NADH oxidized min^−1^ mg protein^−1^ for plasma and liver, respectively [30].

### 5.3. Malondialdehyde (MDA) Concentration

The concentration of MDA (as an indicator of lipid peroxidation following ROS generation) in plasma and post-ischemic heart samples was determined by thiobarbituric acid reactive substances (TBARS) assay. Part of the left ventricle of hearts exposed to I/R protocol from each experimental group was homogenized in a sodium phosphate buffer 10 mmol/L (containing 1 mmol/L ethylenediaminetetraacetic acid and 1 mmol/L butylated hydroxyanisole in 0.15% ethanol), pH = 7.2. The cardiac homogenates (10% *w/v*) or plasma were then used for MDA assessment according to Assimakopoulos [31]. Plasma or cardiac MDA content was determined spectrophotometrically (GENESYS 20 spectrophotometer, Thermo Fisher Scientific), where the absorbance difference A (535–600) was converted to MDA equivalents using the extinction coefficient for MDA 1.55 × 10^5^ M^−1^ cm^−1^. Lipid peroxidation was expressed in µmol/L MDA and nmol MDA mg^−1^ total protein for plasma and heart, respectively.

## 6. Liver Histology

Liver samples, removed from the rat abdominal cavity were flushed with phosphate-buffered saline (PBS; pH: 7.6), fixed in MAW (methanol–acetone–water, 2:2:1), dehydrated in graded ethanol (90% and 100%), cleared in xylene, embedded in paraplast (Sherwood, St. Louis, MO, USA), and serially sectioned at 8 μmol/L. Sections were placed onto Superfrost Plus slides (Menzel-Glaser, Braunschwerg, Germany), deparaffined in xylene, rehydrated in an alcohol gradient, and stained with either hematoxylin and eosin (HE) for a general assessment of the hepatic structure or Sirius red for the detection of collagen fibers and liver fibrosis. The sections were observed under light microscope (Axioscope, Zeiss, Oberkochen, Germany).

## 7. Isolated Heart Perfusion

The animals were heparinized (2.500 U i.m.), anesthetized by an intraperitoneal injection with ethyl carbamate (2 g/kg body weight) and then euthanazied as previously described [32,33]. Hearts were excised, rapidly placed in an ice-cold buffered Krebs–Henseleit solution (KHs) and transferred to the Langendorff apparatus to start the retrograde perfusion through the cannulation of the aorta by a glass cannula. Perfusion was performed at constant temperature (37 °C), pressure (100 mmHg) and flow-rate (12 mL/min) by a modified KHs ((containing, expressed in nmol/L: NaCl 113.0, KCl 4.7, MgSO_4_ 1.2, NaHCO_3_ 25.0, KH_2_PO_4_ 1.2, CaCl_2_ 1.8, glucose 11, mannitol 1.1, Na-pyruvate 5.0 (Sigma-Aldrich, Saint Louis, MO, USA)), constantly oxygenated (95% O_2_ and 5% CO_2_) and kept at pH 7.4 [34]. Cardiac performance was evaluated according to the Langendorff technique as previously described [35,36].

## 8. Basal Conditions

The hearts were allowed to stabilize for 40 min to record the baseline parameters. Inotropism was evaluated in terms of developed left ventricular pressure (dLVP; mmHg, index of contractile activity); cardiac contracture was evaluated in terms of left ventricular end-diastolic pressure (LVEDP), whose baseline value was established at the beginning of perfusion as 5–8 mmHg [37,38].

## 9. Ischemia/Reperfusion (I/R) Protocols

After stabilization, the hearts belonging to each experimental group were subjected to Ischemia/Reperfusion (I/R) protocols, i.e., 30 min of global ischemia followed by 120 min of reperfusion with HKs [37,39]. Cardiac performance was monitored step by step and cardiac parameters were recorded during and at the end of the protocols.

### 9.1. Infarct Size

After the I/R protocols, the hearts were dissected in circumferential sections (2–3 mm) and incubated for 20 min at 37 °C in a 0.1% a buffer solution containing 0.1% nitro blue tetrazolium. The unstained necrotic tissue was separated and weighted, and the infarct size was expressed as a percentage of the total left ventricular mass [40,41].

### 9.2. Cardiac MDA Levels and Protein Oxidation

Homogenate samples (obtained as indicated in the above section) derived from post-ischemic hearts of each experimental group were processed for protein carbonyl content (as an indicator of protein damage following oxidative stress) assessment. Protein oxidation was evaluated by 2.4 dinitrophenylhydrazine (DNPH) assay, following the method of Reznick and Packer (1994) and as previously described [31]. The protein carbonyl concentration (expressed in nmol mg^−1^ protein) was calculated spectrophotometrically (GENESYS 20 spectrophotometer, Thermo Fisher Scientific) by absorbance detected at 375 nm, using the extinction coefficient for DNPH (22 mM^−1^ cm^−1^) and the protein concentration was determined accordingly [31]. Cardiac MDA measurement was performed as previously described.

### 9.3. Western Blot Analysis

Hearts subjected to I/R from each experimental group were homogenized in ice-cold RIPA lysis buffer (Sigma-Aldrich, Saint Louis, MO, USA) containing a mixture of protease and phosphatase inhibitors (1 mmol/L aprotinin, 20 mmol/L phenylmethylsulfonyl fluoride, and 200 mmol/L sodium orthovanadate). Samples were then centrifuged at 15,000× *g* at 4 °C for 25 min; supernatant were collected and the protein concentration was determined using the Bradford method according to the manufacturer’s instructions (Sigma-Aldrich, Saint Louis, USA). Equal amounts of proteins (50 μg) were separated on 10% SDS-PAGE, transferred to a PVDF membrane (RPN303F, GE Healthcare), blocked with nonfat dry milk and incubated overnight at 4 °C with primary antibodies (diluted 1:1000) against p-Akt (Santa Cruz Biotechnology, Dallas, TX, USA) and p-AMPK (Cell Signaling Technology, Danvers, MA, USA); total Akt (Santa Cruz Biotechnology) and AMPK (Cell Signaling Technology) primary antibodies were used as loading controls. The membranes were then incubated for 1 h at room temperature with a horseradish peroxidase (HRP)-conjugated anti-rabbit secondary antibody (diluted 1:2000). Blots were subsequently detected by the enhanced chemiluminescence system (GE Healthcare, Milan, Italy) and densitometric analysis of the bands was performed after digitalization. The areas and the pixel intensity, represented by 256 gray values (0 = white; 256 = black), were evaluated; the background was subtracted. NIH IMAGE 1.6 was used (National Institutes of Health, Bethesda, MD, USA).

### 9.4. Statistics

Statistical analysis was performed using the GraphPad Prism Software ^®^ (version 5.0; Graph Pad Software, San Diego, CA, USA). Data were expressed as the mean ± SEM. One-way ANOVA and the non-parametric Newman–Keuls multiple comparison test (for post-ANOVA comparisons) and Bonferroni multiple comparison test were used. Values of * *p* ≤ 0.05, ** *p* ≤ 0.01, *** *p* ≤ 0.001 were considered statistically significant.

## 10. Results

### 10.1. Rheological Properties of Puddings

**Figure 1:**
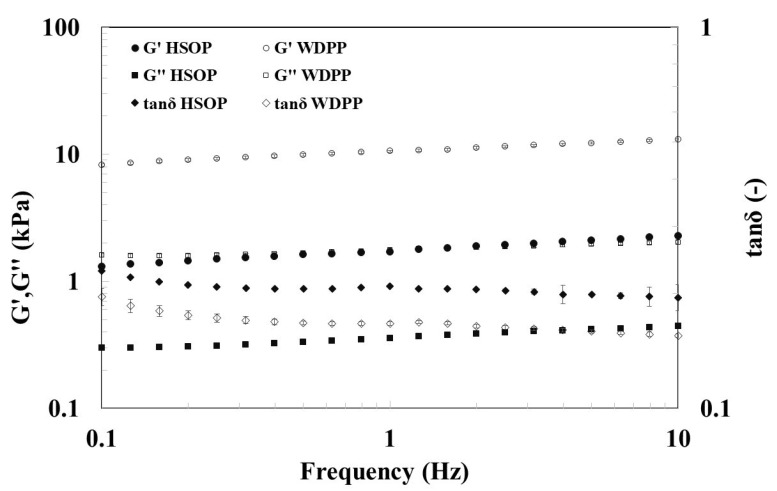
Frequency sweep test at 20 °C of samples HSOP (filled symbols) and WDPP (empty symbols).

Investigations based on small amplitude oscillation tests were carried out to characterize HSOP and WDPP (composition in Table 1). Their recipe was formulated to guarantee that puddings could satisfy the REGULATION (EC) No. 1924/2006 of the European Parliament and Council (20 December 2006), on nutrition and health claims made on food. According to the regulation, the claim “source of protein” for a food can be placed on the label only if at least 12% of its energy value derives from proteins, whereas the claim “high protein content” can be used if at least 20% of the energy value is provided by proteins (as in the case of sample HSOP for which the energy value given by proteins is about 20% of the total, whereas for sample WDPP it is about 33%). Figure 1 shows the frequency sweep tests of both the samples.

The test reported in Figure 1 shows that both the samples are typical weak gels [42]; G’ is higher than G’’ of one order of magnitude and both moduli increase with the frequency of oscillation. Moreover, sample WDPP shows both dynamic moduli higher than HSOP, being more consistent and structured.

### 10.2. Evaluation of Total Polyphenols Content and Antioxidant Properties

The Folin-Ciocalteu assay is a widely employed method for the determination of total phenolic content. The test is based on electron transfer and the redox reaction occurring between Folin-Ciocalteu reagent and polyphenols results in the formation of a blue phosphotungstic-phosphomolybdenum complex, which is quantified by visible-light spectrophotometry. The total phenolic content of the tested samples was expressed as mg equivalent of catechin per gram of sample (mg eq CA/g) for WDPP, HSOP and HSO these values were equal to 0.92 ± 0.20 mg eq CA/g, 1.18 ± 0.18 mg eq CA/g and 0.34 ± 0.05 mg eq CA/g, respectively.

The antioxidant properties of the three samples were also investigated in terms of scavenger ability towards two coloured stable organic free radicals such as DPPH and ABTS. The first one is a lipophilic radical characterized by an absorption maximum band around 515–528 nm, while the second one is a hydrophilic radical with an absorption maximum band at around 734 nm. The antioxidant properties of SD (Table 2), and of the supplementations WDPP, HSOP, and HSO (Table 3) were evaluated in terms of radical reduction in both the performed assays and all the data were expressed as inhibition percentage.

The results obtained indicated that the combination of hemp seed oil and whey-derived proteins puddings has increased antioxidant properties compared to whey-derived proteins puddings and hemp seed oil alone.

### 10.3. “Anthropometric” Variables

After eight weeks of the diet, no significative difference among the experimental groups was detected in terms of weight, length, waist circumference, BMI, abdominal and epididymal fat mass, liver weight, liver to body ratio, heart weight, and CSI (Table 4). All the groups showed similar food and water intake (data not shown).

## 11. Biochemical Analysis

### 11.1. Metabolic Variables

**Figure 2:**
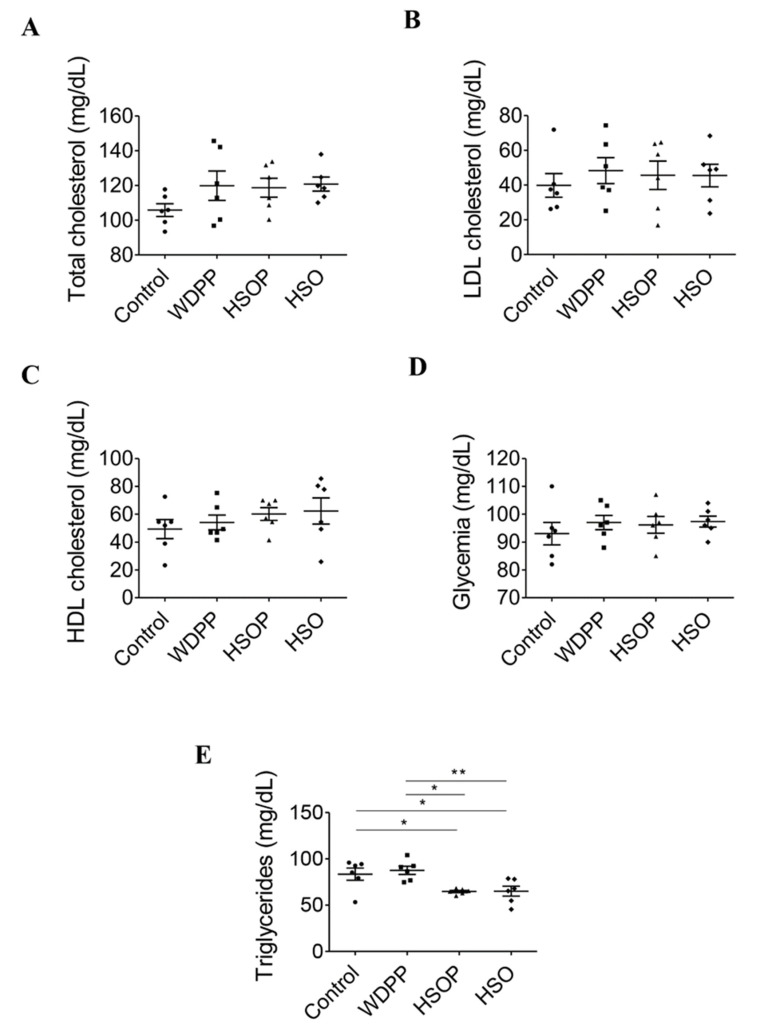
Metabolic plasma variables in rats fed with SD, or WDPP, or HSOP, or HSO for 8 weeks: (**A**) total cholesterol; (**B**) LDL cholesterol; (**C**) HDL cholesterol; (**D**) glycemia; (**E**) triglycerides. *n* = 6 for each group; data are expressed as means ± SEM; statistical significance: * *p* < 0.05, ** *p* < 0.01 (One-way ANOVA and the non-parametric Newman-Keuls Multiple Comparison Test).

No significative differences were found in plasma total, LDL, and HDL cholesterol (Figure 2A–C) as well as in glycemia (Figure 2D). In contrast, plasma triglycerides resulted to be lower in the HSOP and in the HSO groups compared to both the control and the WDPP group (Figure 2E), while the control and the WDPP groups had a similar tendency (Figure 2E).

### 11.2. Organ Injury and Oxidative Stress

**Figure 3:**
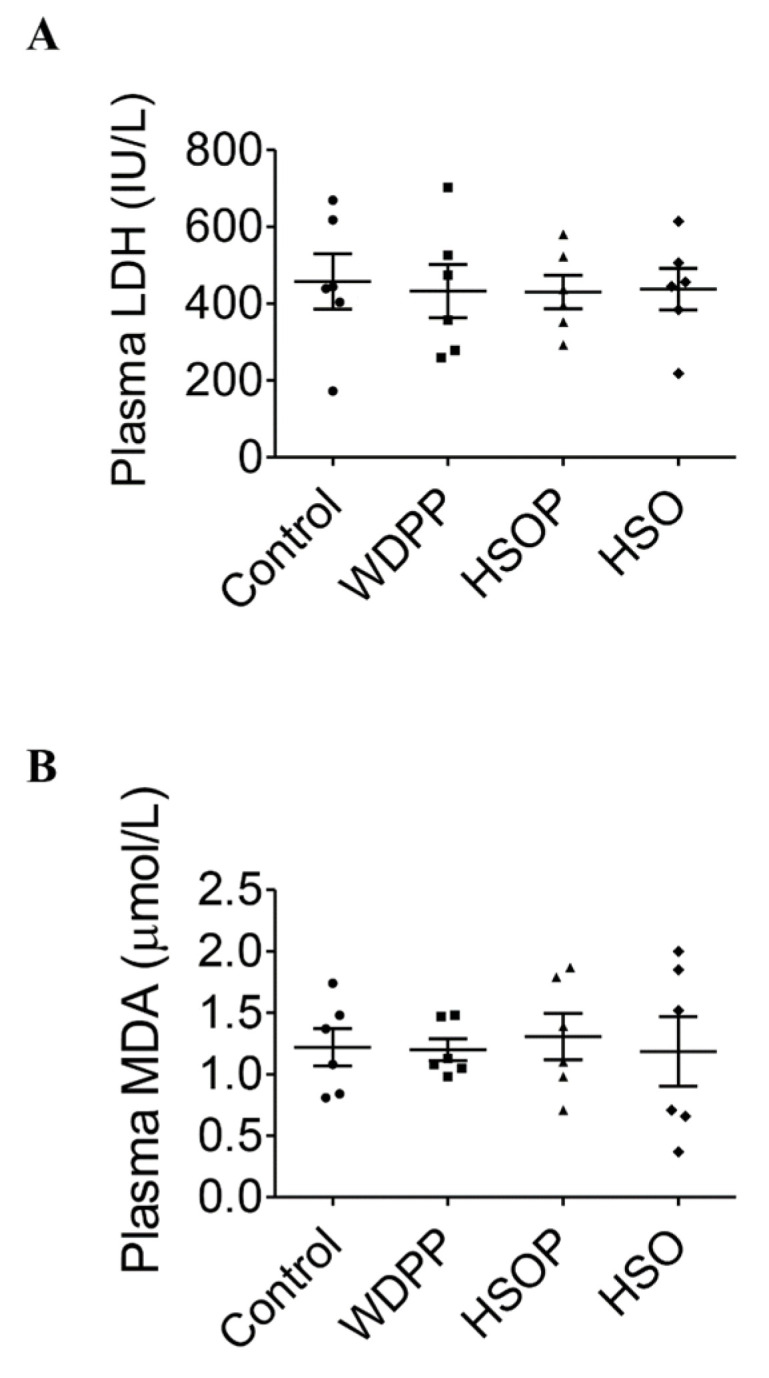
Injury (LDH) and oxidative stress (MDA) markers in plasma of rats fed with SD, or WDPP, or HSOP, or HSO for 8 weeks: (**A**) LDH; (**B**) MDA. *n* = 6 for each group; data are expressed as means ± SEM. statistical significance: One-way ANOVA and the non-parametric Newman-Keuls Multiple Comparison Test.

Plasma LDH (Figure 3A), index of tissue injury, and MDA, index of oxidative stress, did not change in all the experimental groups compared to the SD control group (Figure 3B).

## 12. Liver Histology and Damage

**Figure 4:**
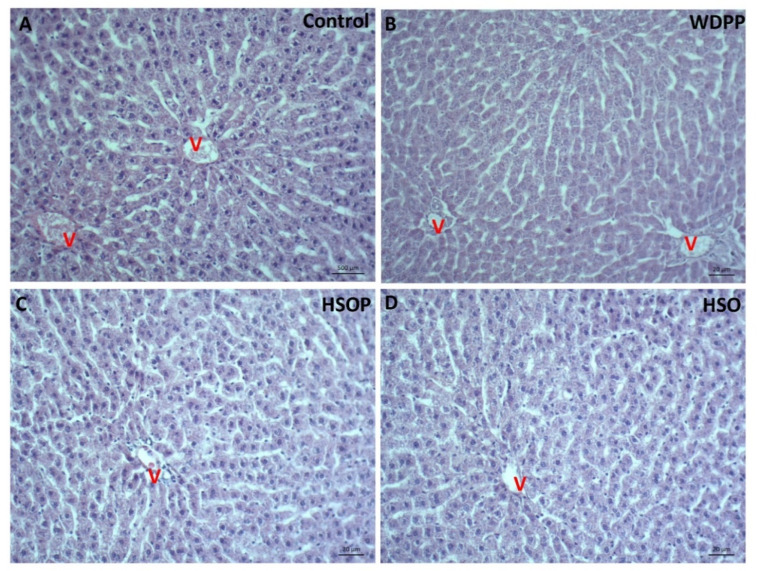
Hematoxylin and eosin (HE) stained liver sections in rats fed for 8 weeks with (**A**) SD; (**B**) WDPP; (**C**) HSOP; (**D**) HSO. V: central vein.

**Figure 5:**
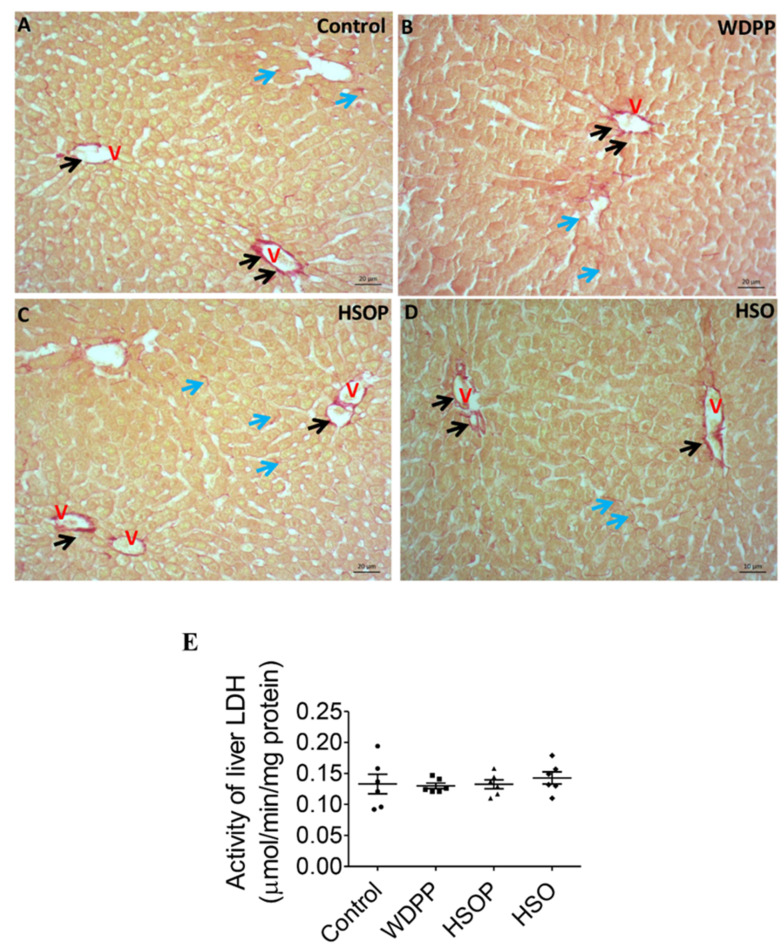
Sirius red-stained liver sections in rats fed for 8 weeks with (**A**) SD; (**B**) WDPP; (**C**) HSOP; (**D**) HSO. V: central vein; black arrows: vessel wall; blue arrows: pericellular spaces. (**E**) LDH activity in the liver of rats fed with SD, or WDP, or WDP + HSO, or HSO for 8 weeks. *n* = 6 for each group; data are expressed as means ± SEM; statistical significance: One-way ANOVA and the non-parametric Newman-Keuls Multiple Comparison Test).

In the HE stained tissue of the control group, liver histology appeared regular, the centro-lobular vein and hepatocytes with radial disposition were evident (Figure 4A). In samples from WDPP (Figure 4B) and HSOP and HSO groups (Figure 4C), the hepatic structure was similar to the control condition and inflammatory process were absent. Parallel sections stained with the Sirius red (Figure 5) showed the presence of collagen fibers localized at the vascular level and in the pericellular spaces in all the experimental conditions (Figure 5A–D).

The LDH activity in the liver was not modified in all the experimental groups (Figure 5E).

## 13. Cardiovascular Function

### 13.1. Basal Cardiac Parameters

Cardiac parameters, obtained after 40 min of stabilization, are detailed in Table 5. Under basal conditions, before the I/R protocol, the hearts from each experimental group did not show significative differences in dLVP, LVEDP, coronary pressure (CP), and heart rate (HR) values (Table 5).

### 13.2. Post-Ischemic Cardiac Function

**Figure 6:**
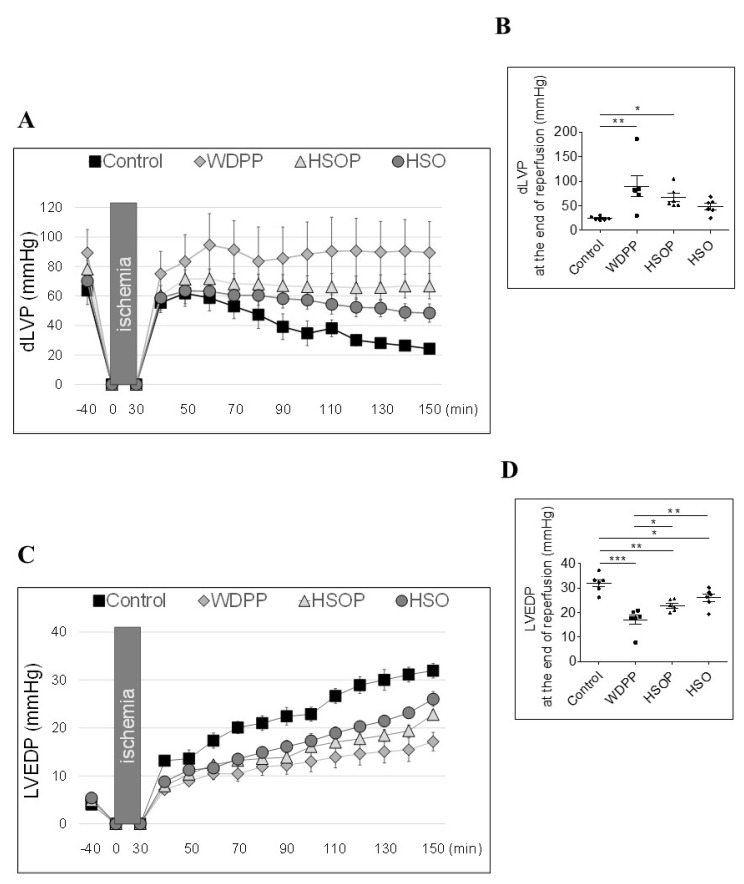
Systolic (**A**) and diastolic (**C**) function under I/R protocols in rats fed with SD, or WDPP, or HSOP, or HSO for 8 weeks. Gray boxes indicate ischemic administration (Bonferroni multiple comparison test). dLVP = 25.3% of total variation between groups (*p* < 0.001); LVEDP = 23.7% of total variation between groups (*p* < 0.001). (**B**,**D**) show dLVP and LVEDP at the end of reperfusion. Data are expressed as changes of dLVP and LVEDP values (millimeters of mercury) from stabilization period to the end of the 120 min of reperfusion with respect to the baseline values for Control, WDPP, HSOP and HSO groups (*n* = 6). * *p* < 0.05, ** *p* < 0.01, *** *p* < 0.001 (1-way ANOVA and Newman-Keuls multiple comparison test).

dLVP represents the systolic function (dLVP; i.e., inotropic activity) and its recovery is an index of cardiac function under and at the end of I/R protocols. In both the postischemic phase (Figure 6A) and at the end of the reperfusion (Figure 6B) the WDPP and the HSOP groups showed a significant recovery of dLVP with respect to the control and the HSO groups. In terms of diastolic function, LVEDP (i.e., contracture state), all the experimental groups showed a reduced contracture with respect to the control, even if it was more pronounced in the WDPP group (Figure 6C,D).

### 13.3. Infarct Size and Cardiac Oxidative Stress

**Figure 7:**
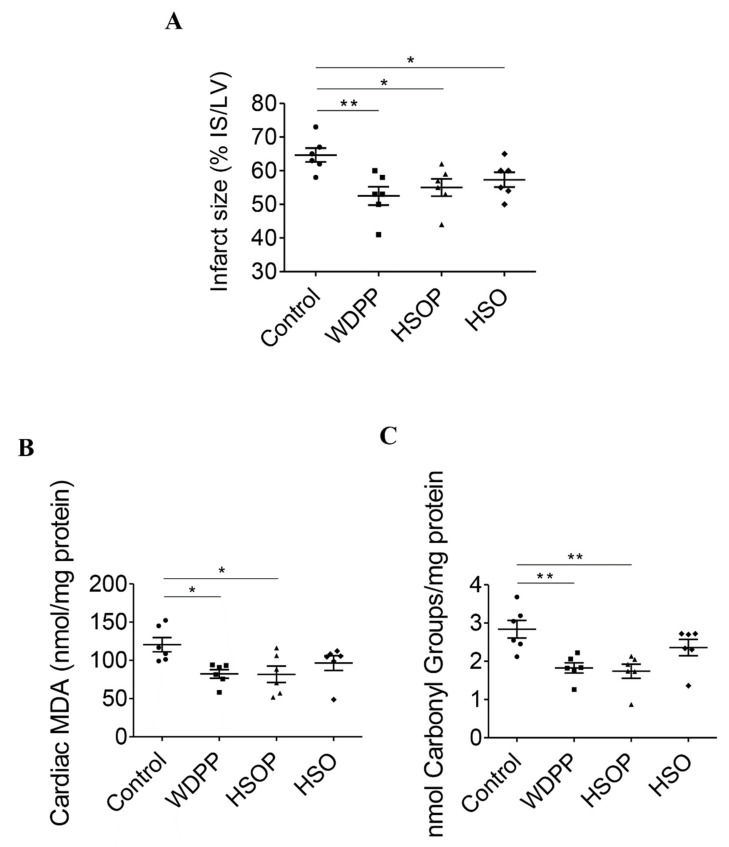
Infarct size (**A**), cardiac MDA (**B**) and cardiac protein carbonyl groups (**C**) in I/R hearts from rats fed with SD, or WDPP, or HSOP, or HSO for 8 weeks. *n* = 6 for each group; data are expressed as means ± SEM; statistical significance: * *p* < 0.05, ** *p* < 0.01 (1-way ANOVA and Newman-Keuls multiple comparison test).

The size of the infarct area after I/R protocols was significantly lower in the WDPP, HSOP, and HSO groups respect to the SD control group (Figure 7A). MDA levels (Figure 7B) and protein carbonyl content (Figure 7C) (used as oxidative stress and protein damage markers), in I/R subjected hearts, were similar in the HSO and in the control group, while in WDPP and HSOP they were significantly decreased.

### 13.4. Western Blot

**Figure 8:**
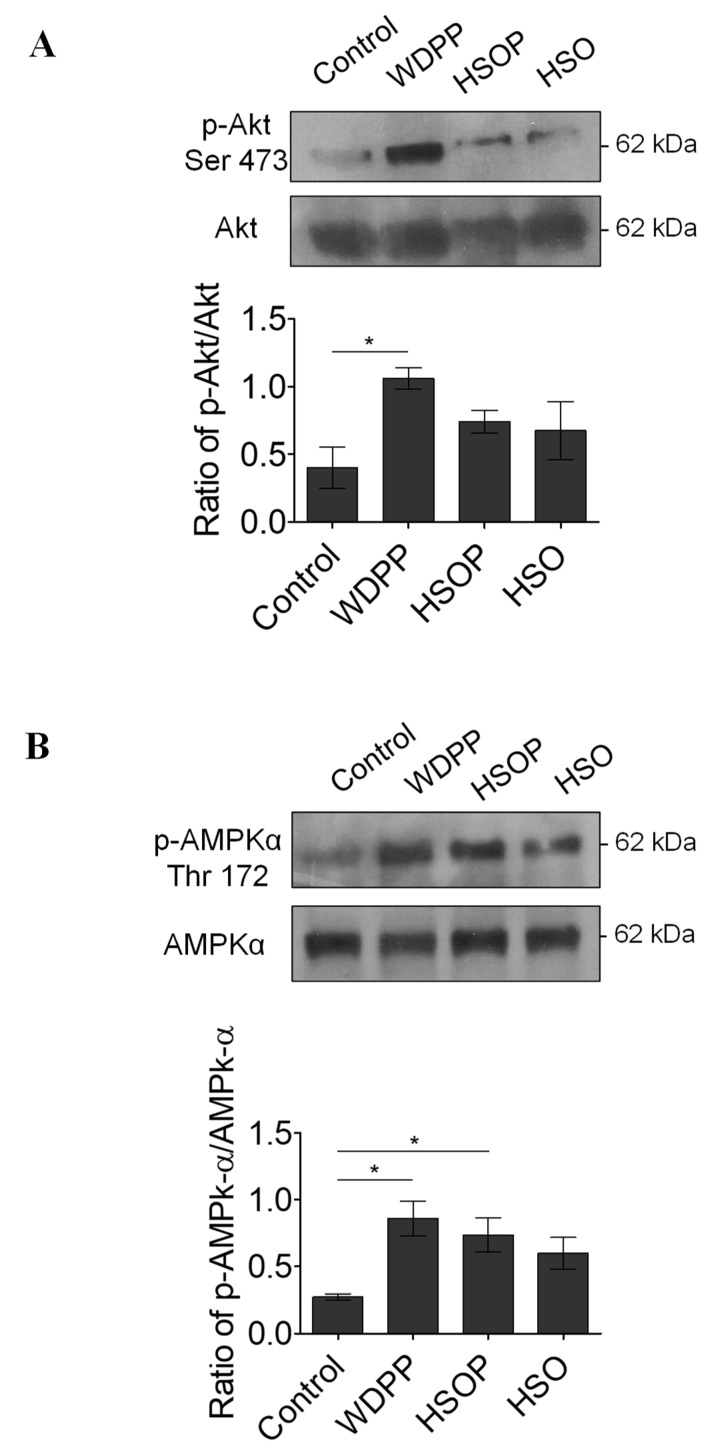
pAkt (**A**) and pAMPK (**B**) levels in I/R hearts from rats fed with SD, or WDPP, or HSOP, or HSO for 8 weeks. *n* = 3 for each group; data are expressed as means ± SEM; statistical significance: * *p* < 0.05. (1-way ANOVA and Newman-Keuls multiple comparison test).

The analysis of Akt (index of the activation of cardioprotective pathways) and AMPK (used as an energy stress sensor and modulator index) phosphorylation levels in I/R hearts revealed that p-Akt increases only in the WDPP group (Figure 8A), and p-AMPK increases in both WDPP and HSOP with respect to the other experimental groups (Figure 8B).

## 14. Discussion

An appropriate nutrition in particularly frail patients can represent an important and non-invasive strategy to counteract the detrimental development of malnutrition and subsequent sarcopenia. In this context, it is well established that dysphagia, owing to swallowing dysfunctions, is one of the major causes of inadequate intake of proteins [43], with consequent impairment of muscle metabolism and function [4]. Since protein intake plays a pivotal role in muscle physiology and homeostasis, a diet supplementation with proteins is proposed to prevent sarcopenia [44]. Thus, in many cases, the incidence of chronic disease may be counteracted by affecting dietary lifestyle. The possibility of using a food-based strategy to influence health positively is very attractive since it may be a very low-cost tool in prevention and treatment. Therefore, researchers and industries are working to identify bioactive nutritional beneficial compounds. In this context, emerging importance is given to functional foods, whose composition includes compounds with beneficial preventive actions and appropriate nutritional effects [20]. Consequently, numerous efforts are currently directed to design and develop functional foods with proper composition and suitable rheological properties able to overcome, in particular, dysphagia-related complications [45]. One of the aims is to draw guidelines to define experimental conditions for testing materials suitable for dysphagic patients [46]. According to Quinchia et al. [47] and Talens et al. [48], typical values of elastic modulus for similar samples characterized as potential foods for patients with dysphagia range between about 0.1 and 1 kPa (at 1 Hz) measured with frequency sweeps at 25 °C (at lower temperatures, these values should slightly increase), whereas the loss tangent should range between roughly 0.1 and 0.5. Thus, it can be suggested that emulgel HSOP obtained by “diluting” sample WDPP with hemp seeds oil (Table 1), are suitable for use as a supplement for patients with dysphagia, and it is confirmed that in the case of emulgels produced with inactive fillers, the addition of the liquid dispersed phase is able to lower the consistency of the continuous one [24,49,50].

In the present study, for the first time, we investigated the possible beneficial effects of a standard diet supplemented with whey-derived proteins pudding (WDPP), or with HSOP (WDPP plus hemp seed oil, HSO), or with HSO alone, using a supplementation produced according to the rheological features suitable for dysphagic-related pathological conditions. We highlighted a significative improvement of cardioprotection in WDPP animals, in terms of both cardiac performance and infarct size, associated with a reduction of oxidative stress and an increase of Akt and AMPK phosphorylation. Even if the lack of a diseased counterpart (a dysphagic and/or malnourished model) may represent a limitation of the study, this work mainly aimed to evaluate the pudding effects on the homeostatic balance of a physiological experimental model, setting the results as a crucial starting point for future analysis.

For a long time, whey was considered as a waste deriving from the milk/cheese industries. However, it has been recently revalued thanks to its positive effects on health [51], owing to the high quality of its protein content [52]. Several studies demonstrated that WDP, which are rapidly digested are able to stimulate muscle protein synthesis with a positive balance on its functionality [51,53]. At the same time, hemp seeds are known to be rich in essential fatty acids able to modulate positively the cardiovascular system [54]. They include an optimal 3:1 ratio of omega 6:omega 3 fatty acids in the form of linoleic acid and alpha-linolenic acid, respectively [54]. This ratio is of interest since omega-3 fatty acids are known to prevent and control heart diseases, while omega-6, when in excess, are unsafe [55].

In our study we did not detect significant differences in the “anthropometric” indices of the different experimental groups, indicating that our supplementations did not interfere with the physiological development of the animals. It is known that WDP and HSO improve metabolic plasma variables [56,57,58]. In line with this evidence, we observed a reduction of triglycerides in the HSOP and HSO groups, with respect to control. This is important since triglycerides are considered not only a risk marker for cardiovascular diseases, but also a possible target for therapeutic interventions [59]. The reduction in triglycerides that we observed in our experimental set may contribute to the cardioprotective effects that will be discussed later in the text.

Of note, food supplementation did not modify significantly plasma LDH and MDA, with respect to the standard diet. An increment in plasma LDH is related to pathological conditions such as cardiovascular alterations, muscle trauma and tissue turnover [60], being indicative of a general damage to the organism. MDA is the main product derived from polyunsaturated fatty acid peroxidation, as a result of oxidative stress targeting biological lipids [61]. Our results suggest that the supplementation proposed in the present study, despite the increase in daily protein or essential fatty acid amount, did not induce tissue damage and oxidative stress. This was supported by data on liver histology and hepatic LDH activity that was similar in all experimental groups.

Importantly, the supplementation with WDPP alone and together with HSO affected the cardioprotective potential of the myocardium against infarct.

I/R protocols are well standardized experimental procedures that allow the evaluation of cardiac recovery and infarct size after an ischemic insult [37], highlighting the possible protective effects of acute and chronic treatments, and of different nutritional states. Under the experimental condition of the present study, maximum protection was reached in the group supplemented with WDPP and, to a lesser extent, in the HSOP group. This was revealed by the significant recovery of cardiac inotropism, the absence of cardiac contracture, and the lower infarct size. After I/R, the excessive generation or accumulation of free radicals can lead to an increased oxidative stress that, in turn, increases lipid peroxidation and protein oxidation [62]. In line with the cardioprotective effects of WDPP and HSOP, our results showed a decrease in both cardiac MDA, and protein carbonylation [63] after I/R. Furthermore, in WDPP an increase of Akt phosphorylation was found, suggesting the activation of important pro-survival cascades typical of cardioprotection under ischemia [30]. Moreover, in both the WDPP and the HSOP groups, ischemic hearts showed an increased activation of AMPK, a crucial orchestrator of cardiomyocytes adaptive response to stress, known to reduce and/or prevent cardiac infarction and contractile dysfunction during I/R [64].

Our data suggest that the protective effects of our puddings may derive from a synergic activity of WDP and HSO. In fact, when HSO is given in association with WDPP, it is effective in inducing cardioprotection, with respect to HSO alone. This may result from the structural unsaturation of HSO that makes it unstable from both a chemical and nutritional point of view, and this may reduce its beneficial properties [65]. At the same time, the antioxidant ability of WDPP [66] may increase the structural stability of HSO, improving its effects when both are given together.

Since ingested whey proteins can be hydrolyzed by gastric and/or pancreatic proteases [67] with the resulting conversion into whey protein hydrolysate, known to possess antihypertensive and cardioprotective abilities [68], we cannot exclude the possibility that this may improve the effects of HSO [69].

## 15. Conclusions

The results of the present study support the possibility that whey, an industrial dairy processing product and an environmental pollutant, is a new source of functional ingredients. Used alone or in combination with HSO in functional foods, it can be an intriguing adjuvant in food-based strategies for patients suffering from inadequate protein intake and the consequent secondary sarcopenia. The limitation of the study is represented by the lack of a diseased/malnourished counterpart to assess the beneficial effects of our supplementations. However, our results represent a promising starting point for future investigations also in a condition of perturbed homeostasis. Since malnutrition and sarcopenia can correlate to cardiovascular dysfunctions and impaired quality of life, a diet supplementation able to increase protein intake, also conferring cardioprotection could be very convenient. Additional investigations are needed to fully understand the potential of WDPP and HSOP in relation to health. An important focus will be represented by the rheological properties of the supplemented food that may make it appropriate for patients with swallowing difficulties. The preliminary results here described propose WDPP and HSOP as non-dangerous functional compounds with putative synergic protective effects and a translational potential in relation to malnutrition and related diseases.

## Figures and Tables

**Table 1:** ID and nutritional values of samples produced with Undenatured Whey Powder Isolate (WDPP), or with Undenatured Whey Powder Isolate plus Hemp Seeds Oil (HSOP), or Hemp Seed Oil (HSO) alone, or standard diet (SD).

		SAMPLE ID	
INGREDIENTS		WDPP (*w/w* %)	HSOP (*w/w* %)
WPI		13.33	13.33
Water		86.67	76.67
Hemp seeds oil		0	9.50
Soy Lecithin		0	0.50
WPI/tot ratio		12	12
Energy (kJ/100 g)		212	556
Energy (kcal/100 g)		50.6	132.8
Energy from proteins (kcal)		50.6	50.6
Total Fats (g/100 g)		0.13	9.9
Saturated Fats (g/100 g)		0.02	0.56
Total Carbohydrates (g/100 g)		0.27	0.28
**SAMPLE ID**		**SAMPLE ID**	
**HSO (per 100 mL)**		**SD (per 100 g)**	
Proteins (g)	0	Proteins (g)	18.6
Carbohydrates (g)	0	Carbohydrates (g)	44.2
Total Fats (g)	99.5	Total Fats (g)	6.2
Saturated Fats (g)	5	Saturated Fats (g)	0.9
Monounsaturated Fats (g)	12.5	Monounsaturated Fats (g)	1.3
Omega-3 Fat (g)	82	Omega-3 Fat (g)	0.3
Omega-6 Fat (g)	64	Omega-6 Fat (g)	3.1
Vitamin (g)	6.1	Vitamin A (IU/g)	15
		Vitamin D_3_ (IU/g)	1.5

**Table 2:** Inhibition percentages of DPPH and ABTS radicals in SD. Data are expressed as means ± STDev (*n* = 3).

	DPPH INHIBITION (%)		ABTS INHIBITION (%)
SAMPLE CONCENTRATION (mg/mL)	SD	SAMPLE CONCENTRATION (mg/mL)	SD
10	14.89 ± 0.99	20	81.92 ± 1.60
15	22.24 ± 1.23	30	83.92 ± 2.67
20	25.65 ± 1.30	40	87.64 ± 0.95
25	36.45 ± 0.65	50	87.87 ± 1.13

**Table 3:** Inhibition percentages of DPPH and ABTS radicals. Data are expressed as means ± STDev (*n* = 3); (One-way ANOVA and the non-parametric Newman-Keuls Multiple Comparison Test). Statistical significance: * *p* < 0.05, ** *p* < 0.01 HSOP and HSO vs WDPP; ^§§^ *p* < 0.01 HSOP vs HSO.

DPPH INHIBITION (%)				ABTS INHIBITION (%)			
SAMPLE CONCENTRATION (mg/mL)	WDPP	HSOP	HSO	SAMPLE CONCENTRATION (mg/mL)	WDPP	HSOP	HSO
10	15.84 ± 1.67	19.64 ± 1.45 ^*,§§^	5.57 ± 0.63 ^**^	20	9.29 ± 1.06	14.08 ± 1.58 ^**,§§^	3.09 ± 0.44 ^**^
15	37.04 ± 1.04	46.8 ± 0.79 ^**,§§^	13.61 ± 1.43 ^**^	30	23.41 ± 1.16	37.49 ± 1.37 ^**,§§^	8.56 ± 0.65 ^**^
20	60.07 ± 2.03	72.62 ± 1.04 ^**,§§^	22.16 ± 1.89 ^**^	40	49.60 ± 0.95	78.47 ± 1.21 ^**,§§^	18.62 ± 1.10 ^**^
25	78.39 ± 0.92	84.33 ± 1.16 ^**,§§^	31.39 ± 1.61 ^**^	50	75.30 ± 1.55	81.66 ± 1.19 ^**,§§^	27.65 ± 0.82 ^**^

**Table 4:** “Antropometric” parameters of rats fed with SD, or WDPP, or HSOP, or HSO for 8 weeks. *n* = 6 for each group; data are expressed as means ± SEM (One-way ANOVA and the non-parametric Newman-Keuls Multiple Comparison Test).

“Anthropometric” Parameters	Control	WDPP	HSOP	HSO
**Food intake (g/die)**	22.5 ± 0.2	21.8 ± 0.3	22.2 ± 0.3	22.0 ± 0.3
**Water intake (mL/die)**	31.7 ± 0.3	33.0 ± 0.3	32.7 ± 0.5	32.5 ± 0.2
**Body weight (g)**	465 ± 17	486 ± 37	469 ± 11	476 ± 8
**Waist circumference (cm)**	22.3 ± 0.3	22 ± 0.5	21.3 ± 0.2	22 ± 0.3
**Body length (cm)**	26 ± 0.1	26 ± 0.2	26 ± 0.2	26 ± 0.2
**BMI (Kg/m^2^)**	7 ± 0.3	7.4 ± 0.6	7.2 ± 0.3	7.2 ± 0.1
**Abdominal fat (g)**	7.1 ± 0.4	7.9 ± 0.3	7.3 ± 0.6	7.5 ± 0.6
**Epididymal fat (g)**	2.6 ± 0.2	3 ± 0.2	3.1 ± 0.3	3.2 ± 0.1
**Liver weight (g)**	14.5 ± 0.4	15.1 ± 0.7	14.7 ± 0.6	14.3 ± 0.9
**Liver to body weight (%)**	3.1 ± 0.13	3.2 ± 0.4	3.2 ± 0.2	3 ± 0.2
**Heart weight (g)**	1.70 ± 0.09	1.88 ± 0.1	1.82 ± 0.09	1.85 ± 0.08
**Cardiac somatic index**	0.37 ± 0.01	0.39 ± 0.03	0.39 ± 0.02	0.39 ± 0.02

**Table 5:** Basal cardiac parameters after stabilization of rats fed with SD, or WDPP, or HSOP, or HSO for 8 weeks. *n* = 6 for each group; data are expressed as means ± SEM. statistical significance: One-way ANOVA and the non-parametric Newman-Keuls Multiple Comparison Test. dLVP, developed Left Ventricular Pressure; LVEDP, Left Ventricular End-Diastolic Pressure; CP, Coronary Pressure; HR, Heart Rate.

Experimental Group	dLVP (mmHg)	LVEDP (mmHg)	HR (Beats/min)	CP (mmHg)
**SD**	64 ± 9	4 ± 0.4	241 ± 23	67 ± 6
**WDPP**	89 ± 16	5 ± 0.9	220 ± 14	61 ± 5
**HSOP**	78 ± 6	5 ± 1	222 ± 13	68 ± 3
**HSO**	70 ± 10	5 ± 1	250 ± 18	66 ± 3
